# A comment on the service-for-prestige theory of leadership

**DOI:** 10.3389/fnhum.2014.00412

**Published:** 2014-06-10

**Authors:** Christopher R. von Rueden

**Affiliations:** Jepson School of Leadership Studies, University of RichmondRichmond, VA, USA

**Keywords:** leadership, prestige, collective action, punishment, cultural anthropology

Successful collective action often depends on the presence of leaders, who bear greater responsibility than other group members for the logistics of coordination, monitoring of effort, and reward and punishment. Leaders may be expected to shoulder more risk, are vulnerable to retaliation from sanctioned group members, and suffer greater reputational damage from failed collective action. What then motivates individuals to be leaders? From an evolutionary perspective, the answer is not straightforward since most of human history occurred in societies lacking significant disparities in material wealth and institutions that grant leaders coercive power. One possibility is that group members share costs by distributing leadership roles over iterations of collective action. However, this is uncommon where inter-individual differences in leadership ability have an impact on collective action. Whether in small-scale egalitarian societies or large-scale stratified societies, group members typically prefer leaders who are superlative in traits such as physical size, knowledge, and prosociality (von Rueden et al., in press).

Price and van Vugt (2014) offer another theoretical solution: followers reciprocate leaders' services by granting them prestige. As a result of their prestige, leaders receive gifts, coalitional support, deference from competitors, or mating opportunity. I have a minor definitional criticism. I do not see prestige as what is conditionally granted to leaders but rather what leaders can automatically produce through their actions: a reputation for delivering benefits to others. What Price and van Vugt (2014) note is that the advantages to prestige may accrue principally during times of need, such as during conflict or food shortage, and thus leadership can act as a form of insurance (Boone and Kessler, 1999).

Since the benefits leaders provide are often public goods, the service-for-prestige theory entails that group members can free-ride by (1) not contributing to collective action, (2) not rewarding leaders, and (3) not punishing group members who fail to reward leaders. This is where the service-for-prestige theory makes unique predictions relative to other theories of leadership: followers will experience punitive sentiment toward other group members who fail to reward effective leaders (or followers will experience prosocial sentiment toward group members who criticize ineffective leaders). Price and van Vugt (2014) present an example from the Ecuadorian Amazon (Price, 2003) where group members who lack respect for popular leaders are themselves disrespected. Future work will need to determine whether such punitive sentiment is sufficient to stabilize group member contributions to leaders, in various cultural and organizational contexts.

Theoretical alternatives to service-for-prestige predict that followers do not experience a collective action problem in bestowing benefits on leaders, because leadership produces private goods not subject to free-riding (costly signaling theory), followers' contributions to leaders are a product of group selection, or leaders recoup their costs by receiving greater *direct* benefits from collective action. Examples of the latter include collective actions that produce goods more beneficial to leaders and their kin (Ruttan and Borgerhoff Mulder, 1999) and leaders who claim a greater share of the spoils (Hooper et al., 2010; Gavrilets and Fortunato, 2014).

As Price and van Vugt (2014) suggest, social neuroscience methods (e.g., identifying the neural correlates of punitive sentiment in public goods games) can help test the explanatory power of the service-for-prestige model against alternative models of leadership. The public goods game has been modified to introduce asymmetries into decision-making over the distribution of public good shares (van der Heijden et al., 2009) or over punishment and reward (O'Gorman et al., 2009). However, caution is required when making inferences from particular experimental games, whose conditions (e.g., player endowments as windfalls) may rarely hold in natural settings or may be interpreted in different ways depending on the cultural context. In highland New Guinea where leaders demonstrated their qualifications via competitive generosity, large offers in the ultimatum game were perceived not as prosocial but as antagonistic (Tracer, 2003).

## Conflict of interest statement

The author declares that the research was conducted in the absence of any commercial or financial relationships that could be construed as a potential conflict of interest.
